# Integrated synteny- and similarity-based inference on the polyploidization–fractionation cycle

**DOI:** 10.1098/rsfs.2020.0059

**Published:** 2021-06-11

**Authors:** Yue Zhang, Zhe Yu, Chunfang Zheng, David Sankoff

**Affiliations:** Department of Mathematics and Statistics, University of Ottawa, Ottawa, Canada K1N 6N5

**Keywords:** whole-genome duplication, fractionation, branching process, evolution, comparative genomics, flowering plants

## Abstract

Whole-genome doubling, tripling or replicating to a greater degree, due to fixation of polyploidization events, is attested in almost all lineages of the flowering plants, recurring in the ancestry of some plants two, three or more times in retracing their history to the earliest angiosperm. This major mechanism in plant genome evolution, which generally appears as instantaneous on the evolutionary time scale, sets in operation a compensatory process called fractionation, the loss of duplicate genes, initially rapid, but continuing at a diminishing rate over millions and tens of millions of years. We study this process by statistically comparing the distribution of duplicate gene pairs as a function of their time of creation through polyploidization, as measured by sequence similarity. The stochastic model that accounts for this distribution, though exceedingly simple, still has too many parameters to be estimated based only on the similarity distribution, while the computational procedures for compiling the distribution from annotated genomic data is heavily biased against earlier polyploidization events—syntenic ‘crumble’. Other parameters, such as the size of the initial gene complement and the ploidy of the various events giving rise to duplicate gene pairs, are even more inaccessible to estimation. Here, we show how the frequency of *unpaired* genes, identified via their embedding in stretches of duplicate pairs, together with previously established constraints among some parameters, adds enormously to the range of successive polyploidization events that can be analysed. This also allows us to estimate the initial gene complement and to correct for the bias due to crumble. We explore the applicability of our methodology to four flowering plant genomes covering a range of different polyploidization histories.

## 1. Introduction

Two orthogonal approaches to the study of fractionation—duplicate gene loss after polyploidization—focus on one hand on the decrease over time of the number of surviving duplicate pairs [1–7] and, on the other hand, the number of syntenically consecutive pairs lost after the event [8–12]. In this paper, we integrate the two in a single model, enabling for the first time inference of all parameters, with wide application to flowering plant genomes.

The basic model of the cycle between whole-genome replication (the result of polyploidization) and fractionation is a discrete-time branching process, reviewed in §2. Each branching event represents a polyploidization, at which time every member of the population gives rise to a variable number of offspring, interpreted as survivors of the fractionation process. Only the current (final) state of the process is observed.

The main theoretical construct is the prediction of the expected number of gene *pairs* (paralogs) generated at each branching event, but only observed at the current time. Grafted onto the branching process model is a way of identifying which of the events gave rise to each gene pair. This is based on a mutational model of gene sequence divergence, causing a decay over time in the similarity between the genes in a pair.

The model enables us to quantitatively account for a major type of comparative genomic data, discussed in §6, the distribution of gene pair similarities in ‘synteny blocks’ (collinear runs of genes on two chromosomes) either within a genome or between two genomes, as can be compiled by methods like SynMap on the CoGe platform [13,14]. For example, based on the parameters of the branching process, we can calculate rates of fractionation after each polyploidization, and examine the extent it varies from species to species, and on whether it is clocklike within genera, families or orders. We have previously applied this approach to flowering plant families that have been affected by more than one polyploidization event over many tens of millions of years: the Brassicaceae [2,3,6], Solanaceae [4], Malvaceae [5,6] and others.

A major limitation, not of the model, but of the previous analyses based on it, is that the distribution of gene pair similarities contains only enough information to estimate one fractionation parameter per branching event, which is not sufficient for most uses. The model, however, also predicts the number of unpaired genes, or singletons, generated by the process at each branching event, which can also be observed in the very same SynMap synteny blocks defined by the gene pairs. As the first novel contribution of this paper presented in §3, we show how these additional data on syntenic structure greatly expand the scope of the analyses based on the branching process model.

The parameters used in synteny block construction are set to control the trade-off between accidental short runs of collinear gene pairs arising through coincidental tandem duplication, non-homologous recombination, gene movement, common domain structure, assembly errors and other factors, on one hand, versus runs genuinely associated with polyploidization events, on the other hand, but shortened over time due to chromosomal rearrangements, individual gene movements and loss of both members of non-essential gene pairs.

These latter processes of erosion over evolutionary time of the number of gene pairs (and, proportionately, of singletons) belonging to blocks, summarized in §5, which may be subsumed under the term ‘block crumble’, can result in severe downward biases in the estimated number of genes affected by early polyploidizations and in the estimation of fractionation rates. To correct this bias, the second innovation of this paper is the introduction of a set of multiplicative constants—crumble coefficients—and a demonstration of how to estimate them.

The archetypical whole gene doubling arises from a tetraploidization event. However, there are many instances of whole-genome tripling and some of higher ‘ploidy’, or multiplicity, in the evolution of the flowering plants. Modelling these cases requires extra parameters. Instead of a single retention probability per event, there will now be two or more. As our third contribution, we reduce the number of parameters to be estimated by elaborating a previous model of retention [15,16] where the number of retained offspring is binomially distributed, conditioned on non-extinction.

With the branching process model in hand, complete with:

— a new calculation of syntenically validated singletons,
— a way of taking into account block crumble, and
— a reduction in parameter number for tripling under a conditioned binomial constraint,

in §6, we illustrate with four flowering plant genomes: poplar (*Populus trichocarpa*), scarlet sage (*Salvia splendens*), durian (*Durio zibethinus*) and black pepper (*Piper nigrum*). Each of these genomes exemplifies a different history of two or three stages of ancient tetraploidy and/or hexaploidy.

## 2. The branching process model

The model, expounded most completely in [4,5], consists of successive branching events at times $t_{1}<\cdots <t_{n-1}$, and observation time *t*_*n*_ > *t*_*n*−1_. The population size, ‘gene complement’, at *t*_*i*_ is *m*_*i*_ but only *m*_*n*_ is observed. At each branching time *t*_*i*_, every member of the population gives rise to some number *j* of offspring, where 1 ≤ *j* ≤ *r*_*i*_, with probability distribution *u*( · ). (In biologically more meaningful terms, every member has exactly *r*_*i*_ offspring and *r*_*i*_ − *j* of these are lost to fractionation.) The replication process corresponds to the concept of ‘2*r*_*i*_-ploidization’, as in tetraploidization (*r*_*i*_ = 2) or hexaploidization (*r*_*i*_ = 3). (Note that while ancient hexaploidy can be inferred for many flowering plants, the process of engendering this state is understood to involve a succession of events, not a single ‘hexaploidization event’.)

The trajectory of the branching process is in effect a sample point from the *n* − 1 probability distributions $u_{1}(i),\ldots ,u_{r_{i}}(i)$ for i=1,…,n−1. There is no provision for *u*_0_(*i*) > 0, for reasons of inference—any model with one or more non-zero *u*_0_(*i*) is the same as some model with all *u*_0_(*i*) = 0 that has the same probability structure on the observations at *t*_*n*_. (For purposes of modelling alone, forgoing empirical application, allowing non-zero *u*_0_(*i*) may be interesting, e.g. for studying limit behaviour. For example, the existing branching/fractionation process is supercritical, but allowing non-zero *u*_0_(*i*) can change this to critical or subcritical.)

Let $a(i)=(a_{1}(i),\ldots ,a_{r_{i}}(i))$ represent the numbers of genes at time *t*_*i*_, with $1,\ldots ,r_{i}$ offspring, so that

**Figure 1.**
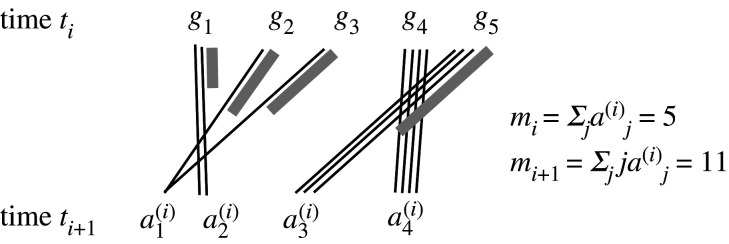
Event with ploidy *r*_*i*_ = 4, showing population of *m*_*i*_ = 5 genes at time *t*_*i*_, each giving rise to 4 progeny, of which 1 ≤ *j* ≤ 4 survive until time *t*_*i*+1_. $a_{j}^{\left(i\right)}$ is the number of times *j* progeny survive. Black lines represent individual progeny that survive, and grey lines represent the total progeny of a gene that do not survive. Here, $a_{1}^{\left(i\right)}=2,a_{2}^{\left(i\right)}=a_{3}^{\left(i\right)}=a_{4}^{\left(i\right)}=1$. From [2].

2.1 $$m_{i}=\sum_{\;j=1}^{r_{i}}a_{j}(i)\;\mathrm{and}\;m_{i+1}=\sum_{\;j=1}^{r_{i}}ja_{j}(i),$$

as in figure 1. Given *m*_*i*_, the probability of **a**(*i*) is

2.2 $$P_{r_{i}}(a(i))=\left(m_{i}a_{1}(i),\ldots ,a_{r_{i}}(i)\right)u_{1}{\left(i\right)}^{a_{1}(i)}\ldots u_{r_{i}}{\left(i\right)}^{a_{r_{i}}(i)}),$$

and the probability of an entire trajectory, defining a paralog gene tree is

2.3 $$P_{r_{1}}(a(1))\ldots P_{r_{n-1}}(a(n-1)),$$

with *m*_1_ ≥ 1 given and the other *m*_*i*_ determined by equation (2.1).

**Figure 2.**
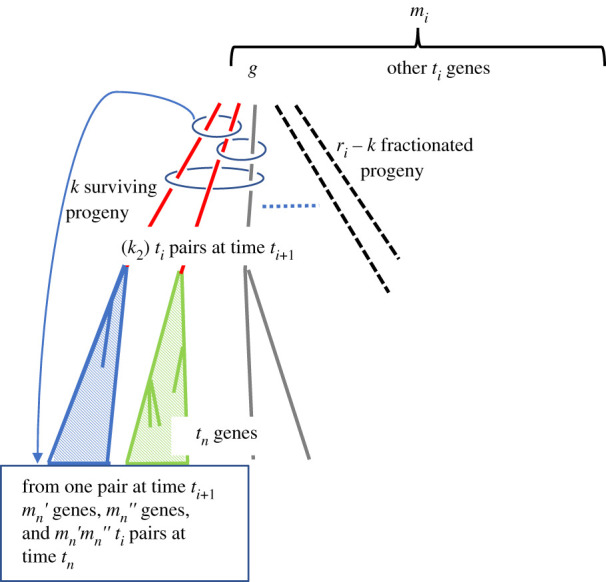
Counting *t*_*i*_-pairs. The three unfractionated progeny of gene *g* define three *t*_*i*_-pairs, as indicated by three ovals. We follow the pair contained in the uppermost oval, as the two members at time *t*_*i*+1_ independently (shaded triangles) evolve into *m*_*n*_′ and *m*_*n*_″ genes, respectively, defining *m*′_*n*_*m*″_*n*_ *t*_*i*_-pairs at time *t*_*n*_. From [2].

Once we know how to calculate these probabilities, it is possible to calculate the **E**(*m*_*i*_). And using the independence of the trajectories starting at any two sibling genes existing at time *t*_*i*_, and their independence from the trajectory between time *t*_1_ and *t*_*i*_, we can calculate **E**(*N*_*i*_) the expected number of pairs of genes at time *t*_*n*_ originating at time *t*_*i*_, as summarized in figure 2.

The accumulation of multinomial coefficients in equations (2.2) and (2.3), and the potentially high degree polynomials might seem computationally formidable. In practice, however, the *r*_*i*_ are generally 2 or 3. Thus individual instances of the model are generally computationally tractable.

For example, suppose there is just *m*_1_ = 1 gene at time *t*_1_, and suppose all *r*_*i*_ = 2. We can write $u(i)=u_{2}(i),$ i=1,…,n−1 for the probability that both progeny of a gene at time *t*_*i*_ survive until time *t*_*i*+1_. We have previously shown [4] the expected number *N*_*i*_ of duplicate pairs of genes born at time *t*_*i*_ and observed at *t*_*n*_ is

2.4 $$\begin{array}{rl} & E(N_{1})=m_{1}u(1)\Pi_{\;j=2}^{n-1}{\left(1+u(j)\right)}^{2}\\ & E(N_{i})=\Pi_{\;j=1}^{i-1}(1+u(j))m_{1}u(i)\Pi_{\;j=i+1}^{n-1}{\left(1+u(j)\right)}^{2}\\ \mathrm{and}\;\; & E(N_{n-1})=\Pi_{\;j=1}^{n-2}(1+u(j))m_{1}u(n-1). \end{array}\}$$

There are *n* − 1 parameters in the vector *u*( · ), and *n* − 1 equations in equation (2.4). The presence of an *n*th variable, namely *m*_1_, means that simply solving the system in equation (2.4) by substituting the observed number of pairs for the expectations of the model can only provide relative values for the parameters in *u*( · ), and not absolute values. There is one kind of observable quantity, however, that cannot be derived from the distribution of gene pair similarities, but are nevertheless predicted by the branching process model, namely the number of singleton genes *S*_*i*_ present at each *t*_*i*_:

2.5 $$\begin{array}{rl} E(S_{1}) & =m_{1}(1-u(1))\\ \mathrm{and}\;\;E(S_{i}) & =m_{1}\Pi_{\;j=1}^{i-1}(1+u(j))(1-u(i)). \end{array}\}$$

## 3. Singletons in synteny blocks

The estimation of the fractionation rates, total gene complement sizes and crumble coefficients associated with the *t*_*i*_ depends on accurate values for the means of the *N*_*i*_ and *S*_*i*_ to substitute in equations such as (2.4) and (2.5). For the *N*_*i*_, this is ensured by the analysis of counting the gene pairs in synteny blocks (cf. §6), and calculating the sequence similarity of each pair to determine the appropriate *t*_*i*_. Singletons, on the other hand, by their nature are not comparable to any other gene, and thus would not seem to be directly associated with any *t*_*i*_.

One way to approach the number of singleton genes might be to subtract the number of genes in all *t*_*i*_ pairs from the total number of genes in the genome. Since a gene may be in several pairs, in synteny blocks corresponding to different *t*_*i*_, however, this calculation requires a more detailed data analysis than is possible from the distribution of gene pair similarities alone. More important, relying on the total number of genes in the genome is very misleading, since many or most of these will have been generated in the time elapsed between *t*_*n*−1_ and *t*_*n*_ by gene family expansion, tandem duplications and other processes.

It is the singletons in the synteny blocks, not the genome total minus the paired genes, that we will use here in the inference of retention rates. Because of their association with the pairs in the blocks, we can pinpoint when a singleton was created, from a pair arising at a specific *t*_*i*_. This results in additional independent observations to help in parameter estimation.

**Figure 3.**
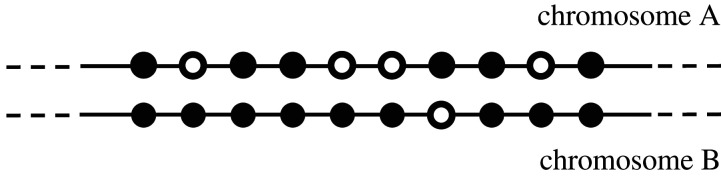
Synteny block on homologous fragments of two chromosomes. Dark circles indicate retained genes, white circles deleted genes. There are five retained gene pairs, four singletons on chromosome B and one singleton on chromosome A.

In the simplest model of fractionation [9], at each step, a random gene pair is selected to lose one member. In a competing class of models [8], gene loss is effected by excision of a variable length fragment of a chromosome, often formulated in terms of a gamma distribution. The study of the internal structure of syntenic blocks, illustrated in figure 3, arose as an indirect way of determining whether fractionation is basically ‘functional’ or ‘structural’. The former posits that fractionation targets specific gene pairs, inactivating or deleting one member of one pair, to redress dosage imbalances or other problems with synthetic or metabolic processes created by whole-genome doubling. The latter, structural explanation represents fractionation as a process of random excision of excess DNA with, say, geometrically distributed length, and which may involve one or more genes, as long as this is not lethal.

Empirically, both types of process play a substantive role [12]. Whatever their relative importance, the expected number of singletons in a synteny block is the sum of the expectations of number of singletons caused by either or both processes.

The number of singletons in a synteny block produced at *t*_*i*_ constitutes the appropriate comparison for the number of pairs in that block, because the singletons were produced by the same branching process as the pairs (or, in the alternative interpretation, during the period between *t*_*i*_ and *t*_*i*+1_).

### 3.1. Synteny and fractionation

Fractionation may affect several duplicate gene pairs in a synteny block at the same time. If this is the case, the loss of one copy or the retention of both is not statistically independent from one gene pair to a neighbouring pair. Since our model only calculates expected values, such non-independence does not matter to the results. However, for future work, such as statistical testing, it is important to understand the relationship between neighbouring gene pairs in their susceptibility to fractionation.

The simplest model would involve each gene pair having the same probability of fractionation, so that one intact pair is chosen at random among the remaining pairs at each step.

Consider the following process. We have an array of *q* 1’s, representing *q* intact duplicate gene pairs. At the first step (*T* = 1), and every subsequent step until *T* = *q*, we pick a 1 at random and transform it to 0, representing the loss by fractionation of one member of that pair.

In [17], we proved the following recurrence for *R*(*T*, *x*), the expected number of runs of 1’s (more precisely, maximal runs) of length *x* at time *T*:

3.1 $$\begin{array}{rl} R(0,q) & =1\\ \mathrm{and}\;\;\;R(0,x) & =0,\;\mathrm{for}\;x\neq q. \end{array}\}$$

Thereafter, for 1 ≤ *T* ≤ *q* − 1 and 1 ≤ *x* < *q* − *T* + 1

3.2 $$\begin{array}{r} R(T,x)=R(T-1,x)-\frac{xR(T-1,x)-2\sum_{i>x}^{q}R(T-1,i)}{q-T+1}. \end{array}$$

This process bears much resemblance to the theory of runs [18] in random binary sequences. Given *q* Bernoulli trials with a probability of success *p* = *T*/*q*, the expected number of successes is *T*, and the expected number of runs of length *x* is *R*(*T*, *x*). However, the variance of the number of successes is non-negligible, whereas it is zero for our process, and the variance of the number of runs of a given length is also greater than our process. Thus our interest in the fractionation process, where the probability of success at each position depends on the total number of successes already achieved.

In [17], we showed how this model was deficient in predicting longer run and gap lengths in the *Coffea arabica* tetraploid genome. We estimated this one gene pair at a time model accounted for about 70% of fractionation events, while a geometric distribution of deletion lengths with mean 3.5 accounted for the remaining 30%.

## 4. Constraints on rates

Under the assumption that the event that each offspring gene is deleted, or survives, is an independent binomial trial, conditioned on at least one such gene surviving, we avoid having to estimate more than one parameter in *u*( · ) for each replication event. The $\Sigma (r_{i}-1)$ ploidy parameters tend to be too numerous when the *r*_*i*_ are larger than 2. As first suggested in [15] and verified in [16], we can circumvent this by assuming gene loss is independent among all the copies, conditional on at least one surviving. For *r*_*i*_ = 3, if *p* is the probability one gene is lost, the probability that

— all three genes survive is (1 − *p*)^3^/(1 − *p*^3^) = *u*′
— two of the three survive is 3*p*(1 − *p*)^2^/(1 − *p*^3^) = *u*
— only one survives is 3*p*^2^(1 − *p*)/(1 − *p*^3^) = 1 − *u* − *u*′.

Let

4.1 $$E=\sqrt{3(3-6u-u^{2})}.$$

Then

4.2 $$u^{\prime }=\frac{u^{2}-(u+1)E+3}{12+2E}\;\mathrm{or}\;u^{\prime }=\frac{u^{2}+(u+1)E+3}{12-2E}.$$

**Figure 4.**
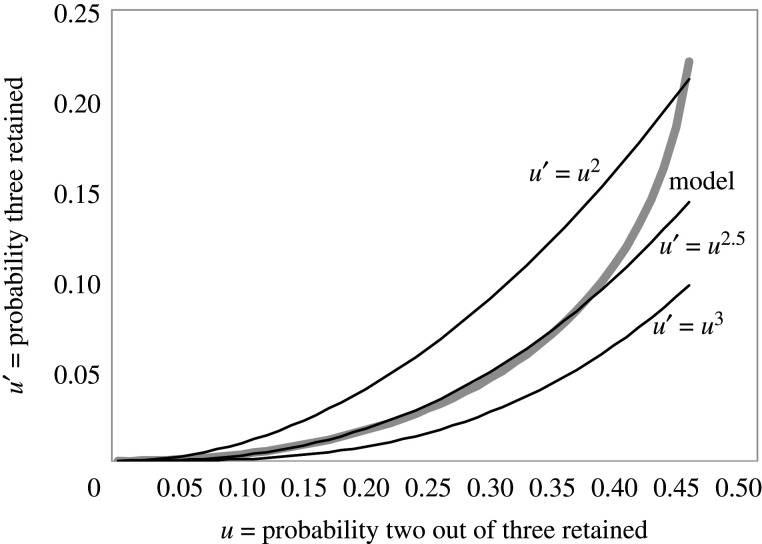
Relationship between *u*′ and *u* based on binomial constraints.

As can be seen in figure 4, this relationship—the left-hand formula in (4.2)—is indistinguishable for practical purposes from *u*′ = *u*^2.5^ as long as *u* < 0.37. While we will not incorporate this constraint into our estimation procedures directly, we will use it to choose among alternative analyses when there are too many parameters compared to equations in the branching process.

## 5. A model for the erosion of synteny blocks over time

The fractionation process has the effect of eroding and completely losing synteny groups over long periods of time, partly because of biological processes like chromosomal rearrangement and gene pair divergence, and partly because of necessary technical limitations on the software detecting the blocks, such as thresholds on minimum amount of collinearity to avoid being swamped by noise.

These latter processes of erosion over evolutionary time of the number of gene pairs (and singletons) belonging to blocks, which may be subsumed under the term ‘block crumble’, can have severe consequences for the inference of retention rates under fractionation. In particular, estimates of *m*_*i*_ are increasingly biased downwards for earlier events, leading to upward biases in the retention rates. In some cases, the estimate of *m*_*i*_ may even be too low to account for all the pairs and singletons observed at *t*_*i*=1_. This represents a weakness of the model that must be corrected, especially for genomes with multiple replication events. To do this we introduce the notion of ‘syntenic cohort’ and a set of multiplicative constants—crumble coefficients—*c*_1_, …, *c*_*n*−1_ for adjusting the *m*_*i*_, and show how to estimate them.

The consequence of this loss is that the gene complement *m*_*i*_ predicted for *t*_*i*_ is underestimated compared with the numbers reconstructed from the synteny blocks at *t*_*i*+1_. The retention probability is thus overestimated. We find that the introduction of a new parameter allows us to estimate the ‘erosion’ rate and hence to make the gene complement at each *t*_*i*_ comparable.

## 6. Four plant genomes

We explore four genomes with various histories of genome replication. We assume that the historical polyploidy events were correctly established for each species, although we could also find them using the method in [6]. The history determines a number of equations similar to (2.4) linking the fractionation rates to the expected values of singletons and pairs observed from each event.

The construction of datasets for our analysis, embodied in software such as SynMap applied to genomes available on the CoGe platform [13,14], involves scanning a genome for pairs of similar genes, then searching for runs of collinear such pairs in two different genome locations. Each run, or ‘synteny block’, must contain a preset minimum number of pairs and have no more than a certain number of consecutive unpaired genes. That the level of similarity of the pairs is relatively uniform in a block, together with the collinearity, lends credence to the conclusion that the pairs were all created simultaneously at one of the replication (branching) times *t*_*i*_, both locations inheriting the pre-replication gene order, and that the interspersed singletons are the remnants of fractionated contemporaneous pairs.

In each case, we

— compare the genome to itself, using SynMap with default parameters,
— construct the distribution of similarities of gene pairs in the synteny blocks,
— find the singleton genes embedded in each synteny block,
— decompose the similarity distribution into its component normal distributions, using [19] or similar method, giving means and proportion of data in each component,
— use maximum likelihood to find a cutoff point between the component distributions,
— assign each synteny block to one of the components according to the mean similarity of the pairs in the block,
— count the total number of pairs and singletons in the two components,
— substitute these numbers for their expected values in the equations for the history of the genome, and solve these to estimate the rates in the model.

In our analyses, we use *u*, *u*′, *v*, *v*′, *c* and *m*_1_ to refer to the survival of two or three (if pertinent) copies instead of one after the first polyploidization event, the survival of two or three (if pertinent) copies instead of one after the second polyploidization event, the crumble constant and the initial gene complement size, respectively.

Note that although our theoretical discussions in §§2, 3 and 5 were phrased in terms of the branching times *t*_*i*_, the equations describing the individual models involved only the *u*( · ), which are really retention probabilities, not fractionation rates. In the following examples, the term *t*_*i*_ serves basically as a label for the *i*th branching event.

### 6.1. Black pepper (*Piper nigrum*)

**Figure 5.**
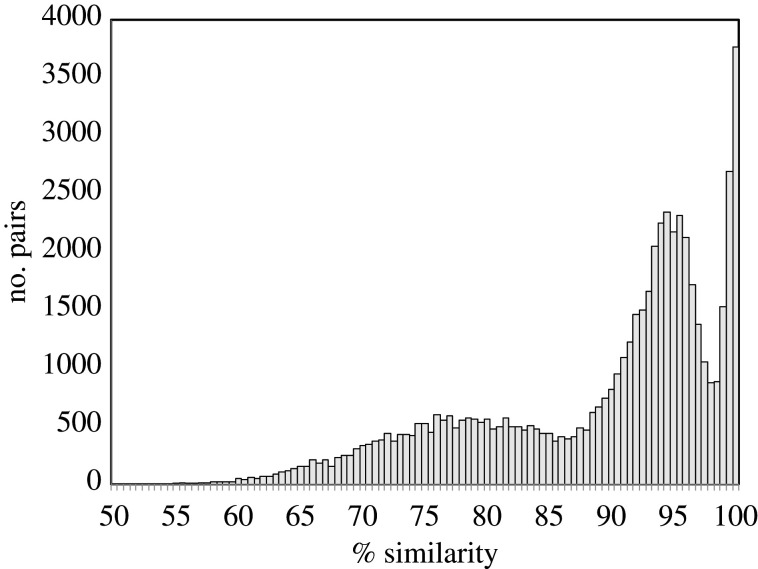
Distribution of sequence similarity of duplicate gene pairs in the black pepper genome.

We choose to analyse the black pepper genome (CoGe ID 56158) since it has undergone the simplest series of whole-genome replications, namely two successive doublings. As a magnoliid, it diverged from the eudicots before the ‘gamma’ whole-genome tripling common to our four other examples in this section. The original report [20] only suggested one doubling event, but the distribution of duplicate genes in synteny blocks in figure 5 is indicative of two, with mean values around 78% and 94%. Additional duplicate pairs closer to 100% similarity may reflect the segmental duplications or high heterozygosity mentioned by the authors, or simply local assembly issues.

The equations where we substitute observed values for expected ones in expressions deriving from the branching process model include those for pairs (cf. equation (2.4)) plus those for singletons (cf. equation (2.5)), as in table 1.

**Table 1.** Equations for rates *u* and *v*, initial population *m*_1_ and crumble *c* for two successive doublings.

event	observed	expected number
*t*_1_	pairs	*cm*_1_*u*(1 + *v*)^2^
*t*_2_	pairs	*m*_1_(1 + *u*)*v*
*t*_1_	singletons	*cm*_1_(1 − *u*)
*t*_2_	singletons	*m*_1_(1 + *u*)(1 − *v*)

To take into account the syntenic crumble process, we repeated the SynMap search for synteny blocks with three different values of the minimum block size parameter: 5 (the default), 4 and 3. The results in table 2 confirm this effect, with over 70% more *t*_1_ pairs and 18% more singletons when the block size criterion is relaxed from 5 to 3. This is substantial, even allowing for some noise with the less stringent criterion. The crumble constant, which estimates the loss of synteny due solely to the block size criterion, is moderate for size 5 and 4, and undetectable for size 3 (*c* ≈ 1).

**Table 2.** Statistics and parameter estimates for the black pepper genome.

block		*t*_1_	*t*_2_	*t*_1_	*t*_2_				
length	cutoff	pairs	pairs	singles	singles	*c*	*u*	*v*	*m*_1_
≥3	89.4%	18 898	15 646	23 637	23 206	1.09	0.29	0.40	30 446
≥4	89.3%	13 593	14 244	19 875	22 773	0.92	0.26	0.38	29 311
≥5	89.1%	11 067	13 711	19 995	23 657	0.85	0.23	0.37	30 417

Of note is the stability of the estimates of *m*_1_, the number of genes in the genome before *t*_1_. Also, the cutoff between the two components of the distribution does not vary, suggesting that the additional gene pairs generated by the less stringent criterion come from the same two events as with the default configuration.

### 6.2. Poplar (*Populus trichocarpa*)

Poplar (CoGe ID 25127) descends from the important whole-genome tripling (known as ‘gamma’) at the origin of the core eudicots. As a member of the Salicaceae family, it has undergone a further whole-genome doubling [21] (the ‘Salicoid’ doubling). The equations for a tripling followed by a doubling are given in table 3.

**Table 3.** Equations for rates, initial population and crumble for a tripling followed by a doubling.

event	observed	expected number
*t*_1_	pairs	*cm*_1_(*u* + 3*u*′)(1 + *v*)^2^
*t*_2_	pairs	*m*_1_(1 + 2*u*′ + *u*)*v*
*t*_1_	singletons	*cm*_1_(1 − *u* − *u*′)
*t*_2_	singletons	*m*_1_(1 + 2*u*′ + *u*)(1 − *v*)

**Figure 6.**
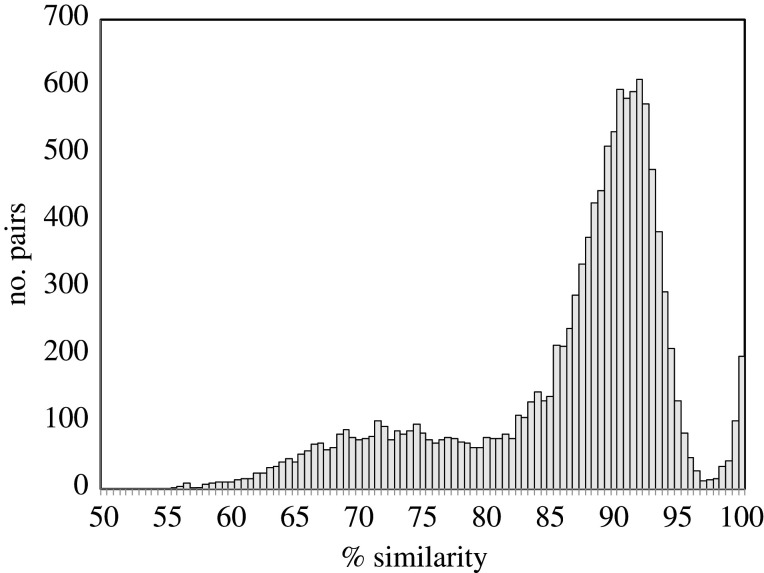
Distribution of sequence similarity of duplicate gene pairs in the poplar genome.

Figure 6 shows a clear separation between the gene pairs created by the two events.

In contrast to the black pepper analysis, we now have more parameters (five) to determine, with only four equations. Here, we make use of the constraint derived from the conditioned binomial analysis developed in §4. Rather than enter the constraint as an additional equation, which would lend it too much weight in simultaneously solving for the other parameters, we simply solved the four equations for a range of values of *c*, namely each value between 0 and 1, in steps of 0.01. Then we picked out the value of *c* that resulted in the closest match to equation (4.2).

Table 4 again shows the stability of *m*_1_ and the cutoff between the two components, despite the 60% increase in the number of *t*_1_ pairs and 32% increase in the singletons when the block stringency is reduced, due to the use of the crumble constant.

**Table 4.** Statistics and parameter estimates for the poplar genome.

block		*t*_1_	*t*_2_	*t*_1_	*t*_2_					
length	cutoff	pairs	pairs	singles	singles	*c*	*u*	*u*′	*v*	*m*_1_
≥3	84.4%	6410	9474	7776	12 810	0.60	0.23	0.02	0.43	17 422
≥4	84.4%	4918	9073	6689	12 749	0.50	0.20	0.03	0.42	17 316
≥5	84.5%	3999	8840	5912	12 726	0.44	0.21	0.02	0.41	17 332

### 6.3. Durian (*Durio zibethinus*)

**Figure 7.**
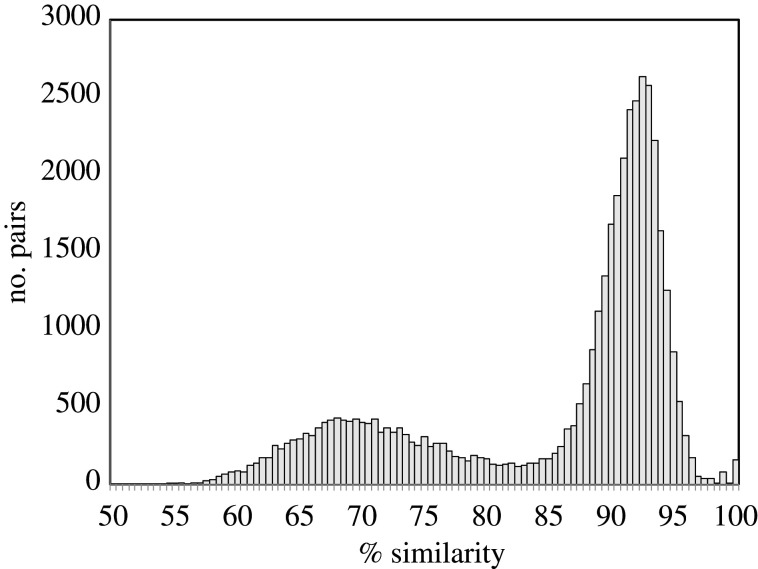
Distribution of sequence similarity of duplicate gene pairs in the durian genome.

When first sequenced the durian genome (CoGe ID 51764) was thought to have undergone a further doubling after the gamma tripling [22]. Subsequent work by ourselves [5] and others [23] showed that the second event was clearly also a tripling (figure 7).

In the case of two triplings, there are still only four equations based on the similarity distribution, two for the pairs, and two for the singletons. But now there are six parameters to find: *u*, *u*′, *v*, *v*′, *c* and *m*_1_. Again, we relied on the conditioned binomial model for the relationship between the two-copy and three-copy survival parameters. We defined a two-dimensional grid for *m*_1_ from 10 000 to 30 000 in steps of 100, and *c* from 0 to 1 in steps of 0.01, and solved the equations for each point on the grid. We then retained all the combinations that closely approximated the constraint in equation (4.2) between *u* and *u*′. Among these solutions, we then chose the one for which *v* and *v*′ also best satisfied this constraint.

In the results in tables 5 and 6, we see stability in the survival rates and *m*_1_, despite the 71% increase in the number of pairs and 23% rise in the number of singletons as the bar is lowered for minimum block length.

**Table 5.** Equations for rates, initial population and crumble for a tripling followed by a another tripling.

event	observed	expected number
*t*_1_	pairs	*cm*_1_(*u* + 3*u*′)(1 + 2*v*′ + *v*)^2^
*t*_2_	pairs	*m*_1_(1 + 2*u*′ + *u*)(*v* + 3*v*′)
*t*_1_	singletons	*cm*_1_(1 − *u* − *u*′)
*t*_2_	singletons	*m*_1_(1 + 2*u*′ + *u*)(1 − *v* − *v*′)

**Table 6.** Statistics and parameter estimates for the durian genome.

block		*t*_1_	*t*_2_	*t*_1_	*t*_2_						
length	cutoff	pairs	pairs	singles	singles	*c*	*u*	*u*′	*v*	*v*′	*m*_1_
≥3	85.8%	11 472	14 854	7876	10 109	0.75	0.27	0.04	0.40	0.11	15 200
≥4	85.5%	8081	14 538	6965	10 602	0.60	0.25	0.03	0.39	0.10	16 000
≥5	85.5%	6704	14 242	6419	10 691	0.53	0.23	0.03	0.39	0.10	16 300

### 6.4. Scarlet sage (*Salvia splendens*)

**Figure 8.**
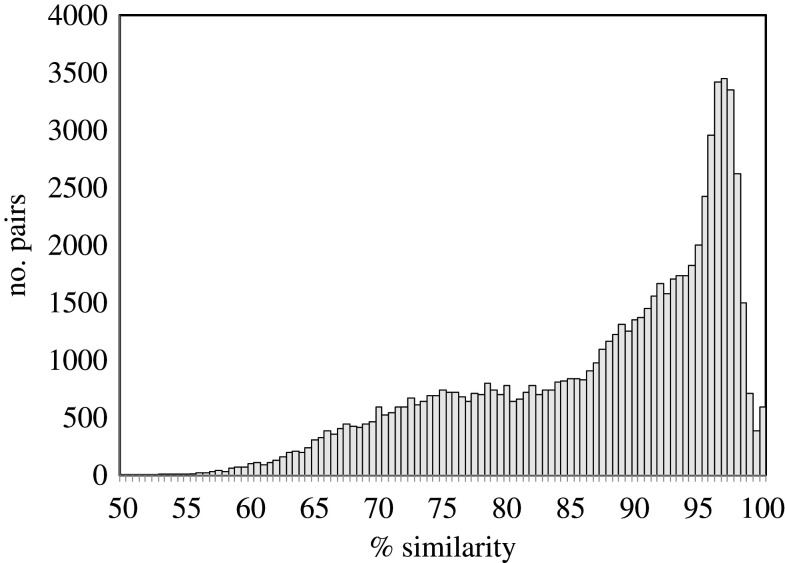
Distribution of sequence similarity of duplicate gene pairs in the scarlet sage genome.

The original report [24] on the scarlet sage genome sequence (CoGe ID 55705) noted a relatively recent whole-genome duplication. Figure 8 shows an earlier event with similarity levels in around 90%, as well as the still earlier gamma tripling event. It is even possible that the apparent gamma component consists of two overlapping parts, but we will not explore the idea of four scarlet sage polyploidization events here (table 9).

For the three events, the first is gamma, a tripling and the third is likely a doubling. The ploidy of the middle event is not clear, and we could not resolve it by the methods of [6]. Thus we will analyse the data in terms of both types of history, two triplings followed by a doubling, represented in table 7, and one tripling followed by two doublings, represented in table 8. In each case, there are two crumble constants, *c*_1_ and *c*_2_, the first covering the period from *t*_1_ to *t*_2_ and the second for the period from *t*_2_ to *t*_3_.

**Table 7.** Equations for rates, initial population and crumble for two successive triplings followed by a doubling.

event	observed	expected number
*t*_1_	pairs	*c*_1_*m*_1_(*u* + 3*u*′)(1 + 2*v*′ + *v*)^2^(1 + *w*)^2^
*t*_2_	pairs	*c*_2_*m*_1_(1 + 2*u*′ + *u*)(*v* + 2*v*′)(1 + *w*)^2^
*t*_3_	pairs	*m*_1_(1 + 2*u*′ + *u*)(1 + 2*v*′ + *v*)*w*
*t*_1_	singletons	*c*_1_*m*_1_(1 − *u* − *u*′)
*t*_2_	singletons	*c*_2_*m*_1_(1 + 2*u*′ + *u*)(1 − *v* − *v*′)
*t*_3_	singletons	*m*_1_(1 + 2*u*′ + *u*)(1 + 2*v*′ + *v*)(1 − *w*)

**Table 8.** Equations for rates, initial population and crumble for a tripling followed by two doublings.

event	observed	expected number
*t*_1_	pairs	*c*_1_*m*_1_(*u* + 3*u*′)(1 + *v*)^2^(1 + *w*)^2^
*t*_2_	pairs	*c*_2_*m*_1_(1 + 2*u*′ + *u*)*v*(1 + *w*)^2^
*t*_3_	pairs	*m*_1_(1 + 2*u*′ + *u*)(1 + *v*)*w*
*t*_1_	singletons	*c*_1_*m*_1_(1 − *u* − *u*′)
*t*_2_	singletons	*c*_2_*m*_1_(1 + 2*u*′ + *u*)(1 − *v*)
*t*_3_	singletons	*m*_1_(1 + 2*u*′ + *u*)(1 + *v*)(1 − *w*)

For the first version of the history of scarlet sage, there are eight parameters, and for the second there are seven. In both cases, there are only six equations. Thus, as in the study of poplar and durian, we recruit the conditioned binomial constraints in §4 to choose among an array of solutions, each a combination of trial values of *c*_1_ and *c*_2_ in a grid array for the first history, and a linear array of *c*_2_ values for the second version. The trial values ranged from 0 to 1 in steps of 0.01.

**Table 9.** Statistics for the scarlet sage genome.

block			*t*_1_	*t*_2_	*t*_3_	*t*_1_	*t*_2_	*t*_3_
length	cutoff 1	cutoff 2	pairs	pairs	pairs	singles	singles	singles
≥3	85%	94%	27 837	15 640	16 801	9941	8632	7726
≥4	84%	94%	18 826	15 628	15 515	9576	9303	8288
≥5	84%	94%	15 265	14 864	14 786	8942	9342	8441

In the first history, two triplings and a doubling, the solutions were assessed to find the combinations of *c*_1_ and *c*_2_ where *u* and *u*′ were close to the predictions of equation (4.2). Among these solutions, we then chose the one where *v* and *v*′ most closely satisfied the same constraint.

For the second history, a tripling and two doublings, the search array involved only *c*_2_, there being enough equations to directly solve the six equations for *u*, *u*′, *v*, *w*, *m*_1_ and *c*_1_.

In tables 10 and 11, we note consistency throughout in the values of *m*_1_, though these are about 15% lower than the values for durian and 22% lower than those for poplar. Given that these are estimates of gene complement before gamma, 120 Ma, the discrepancy is not alarming!

**Table 10.** Parameter estimates for the scarlet sage genome according to the tripling–tripling–doubling model.

block								
length	*c*_1_	*c*_2_	*u*	*u*′	*v*	*v*′	*w*	*m*_1_
≥3	1.1	0.7	0.31	0.02	0.24	0.07	0.69	13 333
≥4	0.9	0.8	0.19	0.03	0.29	0.04	0.65	13 760
≥5	0.8	0.8	0.15	0.04	0.27	0.05	0.64	13 821

**Table 11.** Parameter estimates for the scarlet sage genome according to the tripling–doubling–doubling model.

block							
length	*c*_1_	*c*_2_	*u*	*u*′	*v*	*w*	*m*_1_
≥3	1.06	0.80	0.26	0.03	0.39	0.69	13 297
≥4	0.93	0.87	0.22	0.02	0.38	0.65	13 626
≥5	0.85	0.88	0.21	0.02	0.37	0.64	13 601

Also of note are the identical *w* survival rates in the two histories, and the crumble constants *c*_1_, which are similar.

## 7. Conclusion

We have described a comprehensive account of the similarity distribution of duplicate gene pairs as a function of the time since their creation by whole-genome doubling, as measured by sequence similarity. A branching process model for generating this distribution has too many rate parameters to be estimated based only on the distribution itself. We mitigate this problem by using the frequency of unpaired genes, distinguished from other single-copy genes by their embedding in paralogous synteny blocks, stretches made up largely of duplicate pairs. However, the computational procedures for constructing synteny blocks from annotated genomic data are heavily biased against earlier polyploidization events. We have shown here how to quantify this syntenic ‘crumble’, and how to correct the bias caused by it. Other parameters, such as the size of the initial gene complement, are less accessible. We showed how previously established constraints among some parameters add substantially to the range of successive polyploidization events that can be analysed. In particular, this also allows us to estimate the initial gene complement and helps correct for the bias due to crumble. Finally, we demonstrated the applicability of our methodology to four flowering plant genomes with various doubling and tripling histories.

The importance of singletons in our analysis prompts concerns of whether they may originate, not from fractionation of their paralogs, but from their insertion into one of the homologous chromosomes, such as through the transposon activity rife in plant genomes [25]. However, the major plant transposon families are all well characterized, and transposons are routinely not annotated as genes, and would not show up in the synteny blocks detected by SynMap. Even if the annotation were faulty, masking routines would eliminate transposons, but the genomes we have verified, such as the *Populus* we studied in §6.2, as well as linen (*Linum usitatissimum*) that have unmasked and masked versions of the same assembly in CoGe, show no fewer genes after masking than before. Thus we can be confident in the origin of our singletons in the fractionation process.

Even if we can estimate the retention rates and the gene complement at each event, one critical model parameter cannot be derived from the frequency distribution of gene pair similarities and the number of singletons, namely the ploidy level *r*. Though we may sometimes be able to guess *r* by visual inspection of the output of SynMap, this is not usually the case for earlier events. We have previously shown how to derive additional information from the raw gene pair data in order to construct informative gene triples [6]. Statistics on the configurations of similarities within these triples can then be used to deduce *r*.

## Data accessibility

The annotated genome data used in this paper is freely available on the *CoGe* website. The SynMap program is also available online on that site. The equation solving and other calculations were carried out using the mpl and maxLik packages in R.
